# Effect of Experimental Infection with *Haemonchus contortus* on Parasitological and Local Cellular Responses in Resistant and Susceptible Young Creole Goats

**DOI:** 10.1155/2013/902759

**Published:** 2013-07-11

**Authors:** J. C. Bambou, T. Larcher, W. Ceï, P. J. Dumoulin, N. Mandonnet

**Affiliations:** ^1^INRA, UR143 Unité de Recherches Zootechniques, Domaine Duclos, French West Indies, 97170 Petit-Bourg, France; ^2^UMR703, Ecole Nationale Vétérinaire, Agroalimentaire et de l'Alimentation Nantes-Atlantique, BP 40706, 44307 Nantes, France; ^3^INRA, UE1294 Plateforme Tropicale d'Expérimentation sur l'Animal, Domaine Duclos, French West Indies, 97170 Petit-Bourg, France

## Abstract

This study was carried out to evaluate the relationships of cellular changes in the abomasal mucosa and parasitological parameters, by comparing resistant and susceptible young Creole goats (kids) after experimental infection with *Haemonchus contortus*. The kids were infected over 2 periods (challenges 1 and 2) of 7 and 6 weeks, respectively. Fecal egg count (FEC), blood eosinophilia, packed cell volume (PCV), and body weight were weekly monitored. At the end of both challenges a subgroup of kids was slaughtered for nematode burden measurements and analysis of inflammatory cell infiltration in the abomasal mucosa. The average daily gain was higher in resistant kids after both challenges. Blood eosinophilia and FEC were higher in susceptible kids after both challenges. The number of immature worms and the means of female length were lower after challenge 2 whatever the genetic status. No differences were observed in the eosinophil and mononuclear cell infiltration between challenges 1 and 2 and resistant and susceptible kids. Globule leukocyte infiltration was found higher after the challenge 1 in resistant kids. This effect of the genetic status on globule leukocytes counts but not on the other inflammatory cell highlights the need for further study on the functional activity of these cell populations.

## 1. Introduction

*Haemonchus contortus* is an important gastrointestinal nematode parasite that causes major losses in sheep and goat production worldwide. As anthelmintic resistance is a widespread problem, there is a growing interest in finding alternative control strategies that take advantage of the host's natural resistance [1, 2]. Genetic resistance against gastrointestinal nematode is well described within and between sheep breeds, and to a less extent in some goat breeds [3–7]. Moreover, it is largely admitted that genetic resistance is closely linked to the host immune response [8]. Thus, among alternative control strategies, immunogenetical aspects of gastrointestinal nematode infections in small ruminants appear as promising areas of research. 

 Most of our knowledge of the immune response of small ruminant against gastrointestinal nematode was derived from sheep [9]. It has been shown that genetic resistance is mediated by proliferation mast cells, eosinophils, and globules leukocytes in the abomasal mucosa [10]. The response against gastrointestinal nematode is also associated with increased expression of Th2-type cytokines (e.g., IL-4, IL-5, and IL-13) and parasites-specific immunoglobulin A (IgA) and IgE [11–13]. Despite the similar consequence of gastrointestinal parasitism in goats, few studies have investigated the host response against nematode infection in this model [14]. Some aspects of the host immune response to *H. contortus*, *Trichostrongylus vitrines*, and *Teladorsagia circumcincta* after primary and secondary natural or experimental infection have been studied [15–20]. It seems that the goat immune response against gastrointestinal parasite is less effective than that observed in sheep [21].

 This study was designed to investigate some aspects of the local immune response against *H. contortus* and parasitological parameters comparing resistant and susceptible Creole kids after experimental infection with *H. contortus* third stage larvae (L3). 

## 2. Materials and Methods

### 2.1. Animals and Experimental Design

The study was carried out with a total of 28 growing female Creole kids (15.9 ± 2.5 kg BW; 8-month old) during 2 consecutive periods of 7 weeks for challenge 1 and 6 weeks for challenge 2. All kids were born indoors into a naturally illuminated and ventilated shed at INRA-Domaine Duclos (south of Guadeloupe) and were fed with nematode-free hay. A group of 4 kids (*n* = 2 for challenge 1 and *n* = 2 for challenge 2) was used as uninfected controls for histopathological analysis. There was a lapse of 4 weeks between finishing challenge 1 and starting challenge 2. During the whole experiment, animals received a diet composed of *ad libitum* access to 75-day old *Dichantium *spp. hay and restricted concentrate (100 g/d). In the flock of INRA Guadeloupe, the pedigree of each animal was available since the foundation generation was established in 1979. Fecal samples on relatives and descendants of the experimental kids were regularly collected at 11 months of age under natural mixed infection on pasture conditions for genetic evaluation on the average of 2 FEC measures. Thus, the breeding value for FEC of each experimental kid at 8 months of age was estimated [6]. The 14 resistant and 14 susceptible kids (for each genetic status, *n* = 12 experimentally infected and *n* = 2 control noninfected) initially used in the current study were selected on basis of their extreme breeding value with regard to their cohorts. The resistant and susceptible average predicted breeding values on egg output in a context of natural infection at 11 months of age were distant of 1.04 genetic standard deviation.

On the first day of each challenge and before the morning meal (7.30 h), each kid was individually infected with a single dose of 10 mL of tap water containing 10,000 L3 of *H. contortus*. The kids of the control groups received 10 mL of tap water.

Challenge 1 started with 28 female worm-free (naïve) Creole kids selected on the basis of their genetic status as resistant (*n* = 14; 15.9 ± 1.9 kg BW) or susceptible (*n* = 14; 16.0 ± 3.4 kg BW). After 7 weeks of infection, 8 experimentally infected kids and 2 control noninfected kids were slaughtered (*n* = 5 resistant and *n* = 5 susceptible), the others (*n* = 9 resistant and *n* = 9 susceptible) were drenched with levamisole (Polystrongle, Coophavet, Ancenis, France, 8 mg/kg). Then, kids were housed under worm-free conditions four weeks before the start of the challenge 2. Challenge 2 continued with 18 kids (17.8 ± 2.6 kg BW; 11 months old) (resistant, *n* = 8, 18.3 ± 2.0 kg BW; S, *n* = 8, 17.0 ± 3.0 kg BW) from the initial 28. After 6 weeks of infection, 12 experimentally infected kids and 2 control noninfected kids were slaughtered (*n* = 7 resistant and *n* = 7 susceptible). The selection criterion for slaughter of kids was their FEC values; kids were categorized as low, average, and high FEC.

The L3 of *H. contortus* were obtained 42 days before the challenge. Cultures of feces taken from anthelmintic-susceptible strain were harvested from feces of donor Creole goats monospecifically infected with isolates previously obtained from Creole goats reared on pasture in different farms in Guadeloupe [19]. A standard Baermann procedure was used. After harvesting, L3 were stored at 4°C in tap water at 3000 L3/mL. Each infective dose was suspended in 10 mL of water and was administered orally using a syringe. 

Fecal and blood samples were collected weekly throughout the experiment.

### 2.2. Parasitological Techniques, Blood and Serum Samples

Fecal samples were collected to determine the FEC using a modified McMaster method for rapid determination [19]. Blood samples were collected in EDTA-coated tubes (Becton Dickinson, Plymouth, UK) to measure the number of circulating eosinophils according to the method of Dawkins et al. [22] and the packed cell volume (PCV). Eosinophils were counted using a Malassez cell counter. The PCV was measured using the capillary microhematocrit method.

### 2.3. Worm Counts

For both challenges, kids were necropsied and the abomasum was isolated with its contents. The abomasums were opened along the greater curvature and the contents stored in 5% formalin for total worm counts in 250 mL containers. Each abomasum was then thoroughly washed with warm 0.9% NaCl to detach any adherent nematodes and the washings added to the respective animal's abomasal contents. Parasites were then collected, counted, and sorted according to sex and maturation (immature versus adult). Twenty percent of the total adult female worms from each kid were measured with a calibrated ocular scale.

### 2.4. Histopathology, Immunostaining, and Cell Counting

At necropsy, samples were collected from the tip of the abomasal folds (fundic, body, and pyloric area), fixed 4% paraformaldehyde-phosphate buffer solution, embedded in paraffin wax, and then transversally cut into 5 *μ*m thick sections for histological analysis. Sections were stained using a routine hematoxylin-eosin-safranin staining method. Additional stainings were performed on serial sections of the same samples, including periodic acid-Schiff and toluidine blue. For immunostaining analysis, dewaxing was performed using serial baths in methylcyclohexane and in ethanol solutions of decreasing concentrations (100%, 95%, and 80% by volume in water) of ethanol. After washing in distilled water, antigen retrieval was performed in boiling 10 mM citrate buffer (pH 6.0) for 40 min. Endogenous peroxidase activity was inhibited by incubating in 3% H_2_O_2_ in water for 10 min. The samples were blocked for 20 min in a solution containing 10% normal goat serum, 2% in PBS. The samples were incubated with a CD79 antibody (clone HM57, Dako, Glostrup, Denmark) diluted 1 : 50 in 2% BSA 1 h at 37°C. Unbound primary antibody was removed by washing samples in PBS. Signal amplification and recognition of the primary antibody were performed by incubating the samples with a biotinylated rabbit anti-mouse Ig secondary antibody (Dako) diluted 1 : 300 in PBS/2% BSA. The samples were washed in PBS to remove any unbound secondary antibody and incubated for 30 min at room temperature with the Streptavidin/HRP complex (Dako) diluted 1 : 300 in PBS. Following 3 washes in PBS, the signal was visualised with DAB (Dako). 

The analysis of eosinophils and mononuclear cell infiltration was realized with a semiquantitative grading system. Inflammatory cell infiltration was scored on a four-point scale of 0–3 within thirty microscopic fields randomly selected by skilled pathologists in a double-blind reading manner and lesions were systematically recorded. A score of zero represented no or minimal infiltration, while a score of 3 represented intense infiltration (severe inflammation). A preliminary intraobserver agreement was tested by reproducing this measure 3 times on the same sample to determine the coefficient of reproducibility (CR = 95.3%). Due to the low number of globule leukocytes observed in the abomasal mucosa, a quantitative analysis of this cell population infiltration was performed. A total of thirty microscopic fields were randomly selected in the mucosa and cells were numbered on each sample using a digital camera (Nikon DXM 1200, Champigny, France) combined with an image-analysis software (Nikon Imaging Software). Cellular density was calculated considering total field surface.

### 2.5. Statistical Analysis

The PCV, eosinophilia, FEC, body weight, and parasite burden variables were analyzed by using PROC MIXED of SAS (v. 9.1, SAS Inst. Inc., Cary, NC, USA, 2003) considering the challenges (challenges 1 and 2), the genetic status and their interactions as fixed effects. The FEC and eosinophilia variables were log transformed in order to normalize the variances. For all traits, the experimental unit was considered the kid and was included in the model as a random effect. Comparisons between means were tested by the least squares means procedure with adjustment for multiple comparisons (Tukey-Kramer). The results are presented after backtransformation. The data relative to the semiquantitative analysis of cell infiltration were analyzed by using PROC PROBIT of SAS considering the challenges and the genetic status. Differences of globule leukocyte infiltration of in the abomasal mucosa were assessed via student's paired sample *t*-test. The association between data was determined using Spearman's rank correlation. Significance was declared at ≤5% of probability.

## 3. Results 

### 3.1. Biological, Zootechnical, and Parasitological Measures

**Figure 1:**
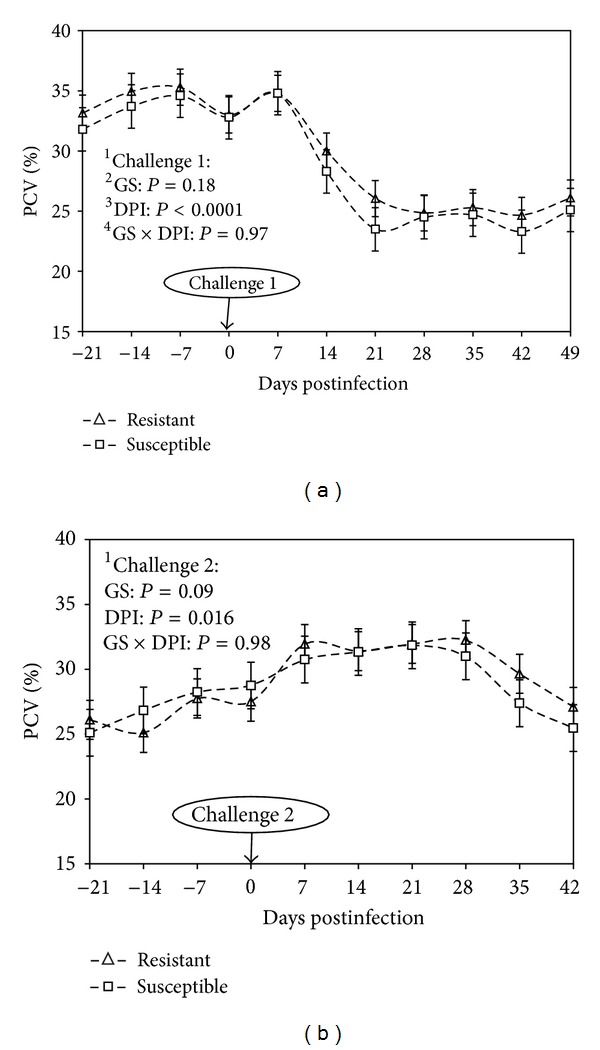
Means of packed cell volume (PCV) comparing resistant and susceptible Creole kids (∆ resistant; □ susceptible) experimentally infected with 10,000 *H. contortus* infective larvae (L3) at day 0 postinfection. ^1^Challenges 1 and 2, first and second challenge with 10,000 *H. contortus* L3. ^2^GS: genetic status against gastrointestinal nematode infection. ^3^DPI: days post-infection, number of days after the animals were infected with 10,000 *H. contortus* L3. ^4^GS ×  DPI: interaction between days post-infection and genetic status.

The PCV values significantly decreased during challenge 1 in resistant and susceptible kids until 28 d.p.i. (days postinfection, *P* < 0.001; Figure 1). A slighter significant decrease was also observed during challenge 2 in both groups from 28 to 35 d.p.i. (*P* = 0.016, Figure 1). No difference was observed between resistant and susceptible kids during both challenges (*P* > 0.05, Figure 1). No interaction between genetic status (GS) and d.p.i. was observed.

**Figure 2:**
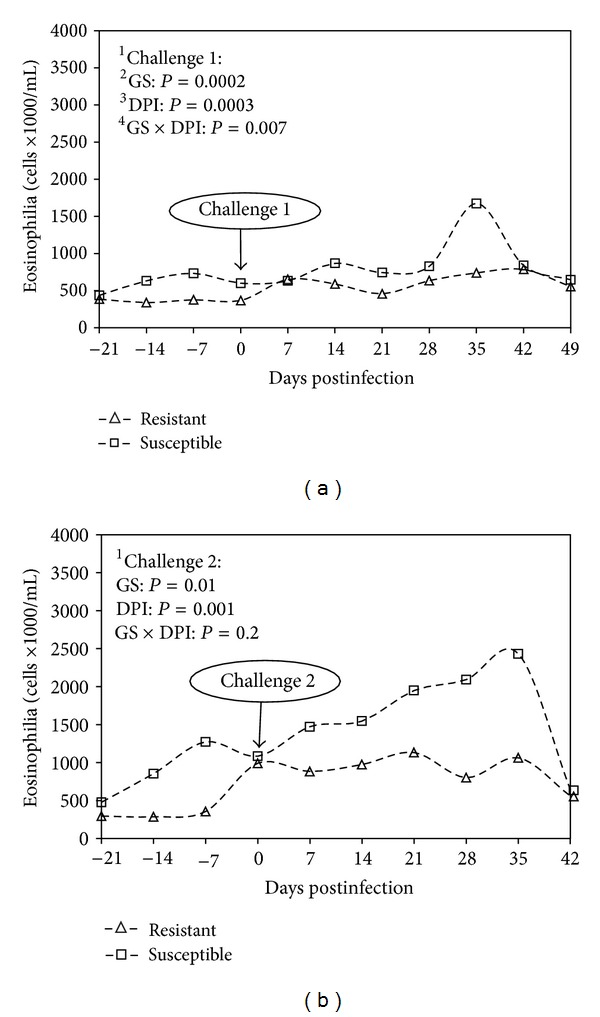
Geometric means of blood eosinophils comparing resistant and susceptible Creole kids (∆ resistant; □ susceptible) experimentally infected with 10,000 *H. contortus* infective larvae (L3) at day 0 postinfection. ^1^Challenges 1 and 2, first and second challenge with 10,000 *H. contortus* L3. ^2^GS: genetic status against gastrointestinal nematode infection. ^3^DPI: days post-infection, number of days after the animals were infected with 10,000 *H. contortus* L3. ^4^GS ×  DPI: interaction between days post-infection and genetic status.

Blood eosinophilia significantly increased during challenge 1 and showed a peak at 35 d.p.i. more pronounced in susceptible kids (Figure 2). Then, during challenge 2 blood eosinophilia increases significantly in both groups with a peak in susceptible kids between 35 and 42 d.p.i. (*P* = 0.001, Figure 2). Blood eosinophilia was significantly higher in susceptible kids compared with the resistant ones during both challenges (*P* = 0.0002 for challenge 1 and *P* = 0.01 for challenge 2, Figure 2). No interaction between GS and d.p.i. was observed.

**Figure 3:**
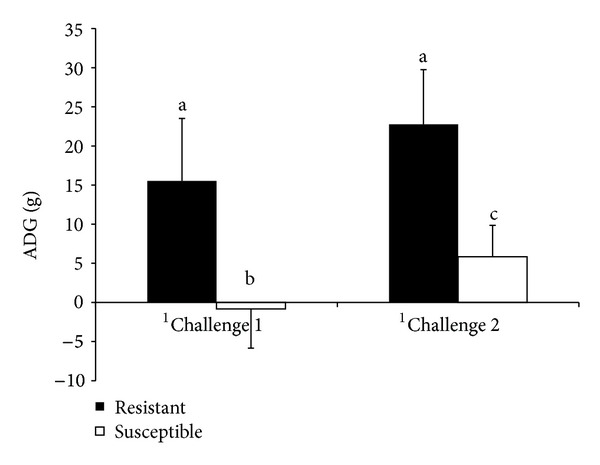
Means of average daily gain (ADG, g) comparing resistant and susceptible Creole kids experimentally infected with 10,000 *H. contortus* infective larvae (L3) at day 0 postinfection. Results are mean ± standard deviation. ^1^Challenges 1 and 2, first and second challenge with 10,000 *H. contortus* L3.

The average daily gain (ADG) was negative in susceptible kids during challenge 1 but not in the resistant ones (Figure 3). During the challenge 2, the ADG in susceptible kids increased to reach a positive value but remains lower than that observed in the resistant kids (Figure 3). 

**Figure 4:**
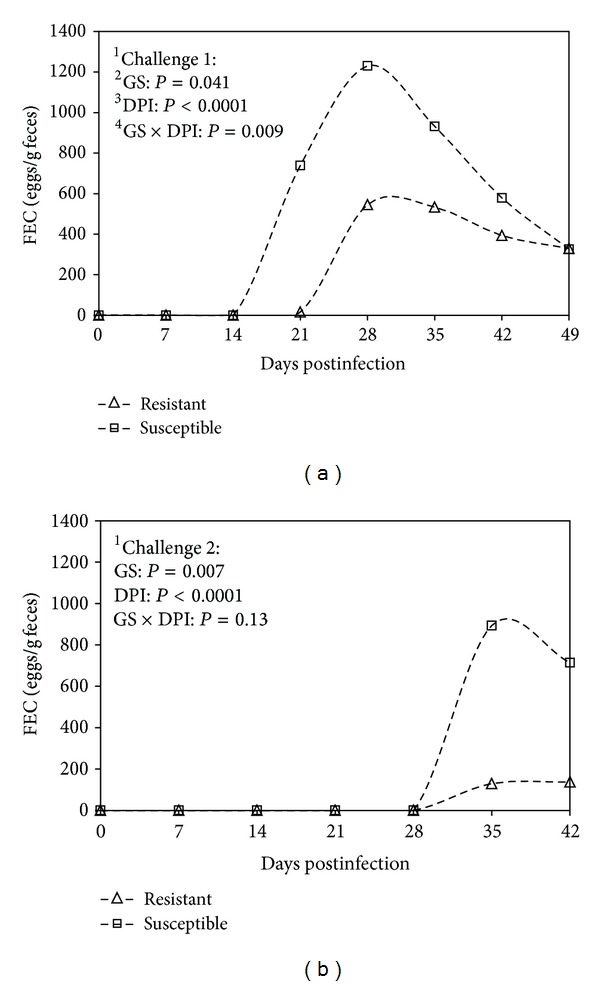
Geometric means of fecal egg count (FEC) comparing resistant and susceptible Creole kids (∆ resistant, □ susceptible) experimentally infected with 10,000 *H. contortus* infective larvae (L3) at day 0 postinfection. ^1^Challenges 1 and 2, first and second challenge with 10,000 *H. contortus* L3. ^2^GS: genetic status against gastrointestinal nematode infection. ^3^DPI: days post-infection, number of days after the animals were infected with 10,000 *H. contortus* L3. ^4^GS ×  DPI: interaction between days postinfection and genetic status.

 The FEC remained at zero until 21 days postinfection during challenge 1 and until 28 d.p.i. during challenge 2 in all groups (Figure 4). A peak in the FEC was observed between 28 and 35 d.p.i. in all groups during challenge 1. The FEC was significantly higher during challenge 1 compared with challenge 2 in all groups (*P* = 0.01, Figure 4). The FEC was higher in susceptible compared with resistant kids during both challenges (*P* < 0.05). A significant interaction between the GS and the d.p.i. was observed during challenge 1 (*P* = 0.009, Figure 4). This interaction was not significant during the challenge 2.

 Postmortem analyses were realized 49 days after the primary infection (challenge 1) and 42 days after the secondary infection (challenge 2). No statistical difference was observed between challenge 1 and challenge 2 for male and female worm counts. Counts of immature male and female worm were higher after challenge 2 at 42 d.p.i. compared with challenge 1 at 49 d.p.i. (*P* = 0.02 and 0.05, resp., Table 1). In contrast, means of female length were higher after challenge 1 compared with challenge 2 (*P* = 0.02, Table 1). No difference between resistant and susceptible kids was observed after both challenges for the different worm counts, except for female length after challenge 1 which showed a tendency, given the number of kids (*n* = 12), for a higher value in susceptible kids (*P* = 0.07, Table 1). In addition, worm female length showed high significant correlation coefficients with FEC (*r* = 0.70, *P* < 0.0001, Table 2) and PCV (*r* = −0.72, *P* < 0.0001, Table 2). A significant positive correlation coefficient was also observed between female worm count and blood eosinophils (*r* = 0.53, *P* < 0.0001, Table 2). However, the correlation coefficient between male worm count and blood eosinophils was negative (*r* = −0.57, *P* < 0.0001, Table 1). 

**Figure 5:**
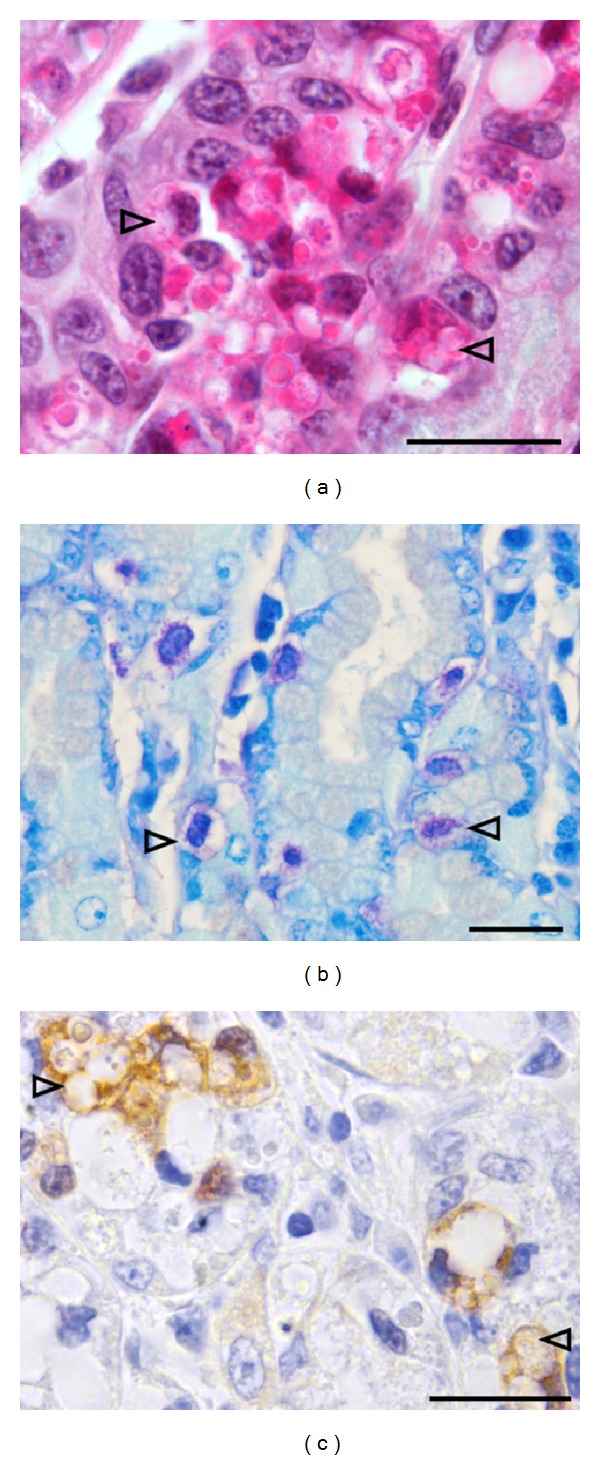
Globules leukocytes identification under light microscopy in the abomasal mucosa of kids experimentally infected with *H. contortus* (examples indicated with arrowhead). (a) Hemalun eosin saffron staining. Globoid cells are rounded, with a round nucleus and abundant cytoplasm filled with eosinophilic material and optically clear vacuoles. (b) Toluidine blue staining. (c) Immunostaining with CD79 antibody. Cytoplasmic signal is noted. Bars = 100 *μ*m.

**Figure 6:**
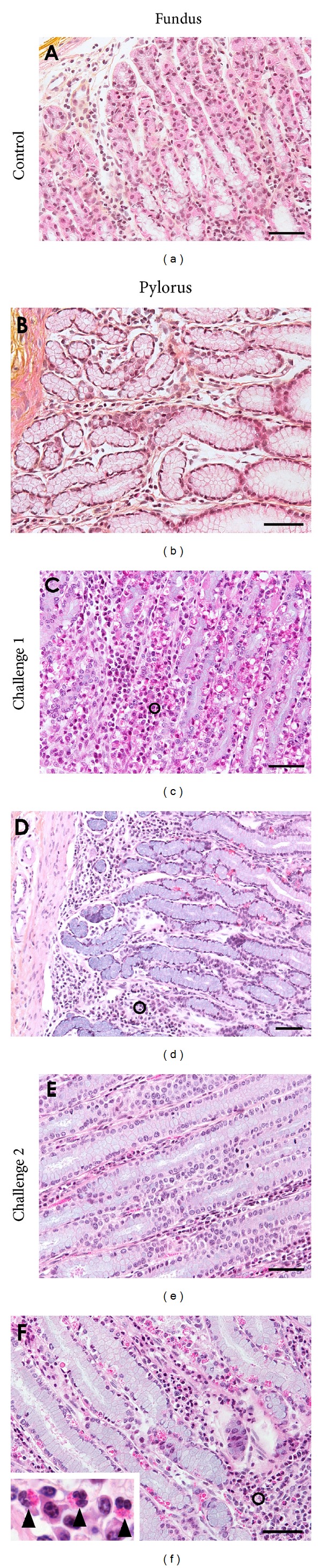
Eosinophils and mononuclear cell infiltration in the fundic and the pyloric abomasal mucosa under light microscopy of Hemalun eosin-stained section of kids experimentally infected with *H. contortus*. ((a) and (b)) Abomasal mucosa of control uninfected kids. A few mononuclear cells are scattered along the connective tissue of the mucosa. ((c) and (d)) Abomasal mucosa at 49 days postinfection of challenge 1, density of cells was multifocally increased in the lamina propria of the mucosa. ((e) and (f)) Abomasal mucosa at 42 days postinfection of challenge 2. Circles (***ο***) show inflammatory cells corresponding mostly to mononuclear and eosinophils cells (see inlet, arrowhead). Bar = 100 *μ*m.

Microscopic evaluation of abomasal samples showed some histological alterations for all infected kids. Some diffuse and marked oedema was noted in the submucosal connective tissue. No alteration of the abomasal mucosa was observed for the control noninfected kids. All along the mucosal epithelium was randomly scattered small cells (around 25 *μ*m) with abundant cytoplasms filled with numerous vacuoles containing hypereosinophilic material or less often optically empty. These cells, so-called globule leukocytes, were metachromatic using toluidine blue staining indicative of their histamine and heparin rich content. Metachromasia of the cytoplasm is responsible for the purple appearance of these cells compared to the blue staining of the others components of the tissue (Figure 5). No other specific staining was positive for them. However, all samples showed a nonspecific CD79 immunoreactivity of some globule leukocytes (Figure 5(c)). Some diffuse inflammatory cell infiltration was also observed in the mucosa and submucosa of all infected kids after both challenges and were mainly composed of eosinophils and mononuclear cells (Figure 6). Globules leukocytes were observed between epithelial cells of gastric glands (Figure 6). After semiquantitative evaluation, no difference between eosinophils and mononuclear cell infiltration intensity was observed (*P* = 0.98, Table 3(a)) after both challenges. A significant decrease of the cellular infiltration intensity was observed after challenge 2 (*P* = 0.041, Table 3(a) and Figure 6). Significant moderate negative correlation coefficients were found between globule leukocyte infiltration in the abomasal mucosa and immature worm burden (*r* = −0.48, *P* = 0.0001). No obvious differences were observed in the eosinophils and mononuclear cell infiltration between challenge 1 and 2 and resistant and susceptible kids (Table 3(a)). In contrast, globule leukocyte infiltration was found higher after the challenge 1 in resistant kids (*P* = 0.04, Table 3(b)) but not after challenge 2.

## 4. Discussion

In this study, we evaluated the effect of primary and secondary experimental infection of susceptible and resistant Creole kids with *H. contortus* on PCV, blood eosinophils, ADG, FEC, worm counts, and abomasal histopathological changes. The primary infection (challenge 1) induced moderate anaemia, whatever the genetic status, and weight loss only in susceptible kids. During the secondary infection (challenge 2), anaemia and weight loss were not observed. This subclinical infection observed during the secondary infection is in accordance with previous results in the same animal model showing that, in goats previously infected by *H. contortus*, a degree of protection occurred [19].

 Blood eosinophilia increased significantly after the primary infection, but no significant increase was observed after the secondary infection. Susceptible kids showed higher blood eosinophilia than the resistant ones. This result is in accordance with our previous data showing a higher blood eosinophilia in susceptible compared with resistant kids previously maintained on pasture then experimentally infected with *H. contortus* [20]. In contrast, in sheep it has been showed that blood eosinophilia was positively correlated with resistance against *T. colubriformis* and *H. contortus* [22–24]. At first sight, it could be suggested that the role of the blood eosinophilic response would be different in goats compared to sheep. However, our data showed that blood eosinophilia at slaughter was negatively correlated with adult worm counts and with female worm length. Moreover, after both challenges female length was numerically lower in resistant animals, particularly after primary infection, but no statistical difference was evidenced found. This is probably due to the high individual variability and the low number of animals per groups. Previous studies in sheep experimentally infected with *H. contortus* showed a high correlation between worm length and fecundity [12, 25]. Our data showed that female length was negatively correlated with PCV and positively correlated with FEC. These results suggest that the lower FEC observed in resistant Creole kids could be due to lower female worm fecundity. 

 After the second infection, the mean of FEC and female length decreased and the prepatent period increased whatever the group, suggesting the development of a more efficient protective response. Moreover, the increase in the number of immature worm suggests that the protective response against *H. contortus* would be expressed early after the secondary infection. Thus, the immune response against *H. contortus* would be also directed against immature worm, contrary to what has been observed in two sheep breeds [26]. However, in our study postmortem analyses were realized 49 days after the primary infection and 42 days after the secondary infection. Owing to the fact that no difference of worm counts was observed between resistant and susceptible kids after the primary infection, we decided to slaughter kids earlier after the secondary infection. Our hypothesis was that a delayed development of worm at the early stage of infection could not be observed later due to the highest probability of rejection of immature worm with time and/or replacement of old adult worm by the moult of immature to adult. Thus, the difference of immature worm counts observed between the primary and the secondary infection could be due to the length of time after infection. 

 Histological examination of the abomasal mucosa showed a more pronounced cellular immune infiltration after the primary infection than after the secondary infection. In contrast, a previous study in *H. contortus* experimentally infected goats showed that the cellular infiltration was significantly more pronounced in reinfected animals compared with the primarily infected one [18]. Nonetheless, whereas in this study animals were reinfected after 30 days of infection without drenching, here animals were drenched after the primary infection and remain parasite-free during a four-week period before the secondary infection. Our results could be also explained by the difference of one week postinfection between the primary and the secondary infections at slaughter. That suggests a late postinfection stage cellular infiltration. In the current study, the intensity of the cellular infiltration was not affected by the genetic status, except for globule leukocyte infiltration which was higher in resistant animals after the primary infection. Moreover, medium negative correlations were observed between globules leukocytes and immature worm burden whatever the genetic status. Previous studies in immunized sheep showed that globule leukocytes were implicated in the immune exclusion of challenge larvae [27–29]. Recently, Robinson et al. (2010) monitored the local cellular immune response in immunized sheep challenged with *H. contortus* and observed a peak of globule leukocyte infiltration in the abomasal mucosa at five days after infection [30]. In accordance with this study, our data suggest that globule leukocytes would be implicated in the response against larval stage of *H. contortus* a mechanism associated with the genetic resistance. In contrast, previous studies on goats showed an abundant globule leukocyte infiltration in the abomasal mucosa over 10 weeks after infection, suggesting that these cells would be associated with adult nematodes rejection [17]. Interestingly, the nonspecific CD79 (marker for B cell) immunoreactivity of globules leukocytes, observed in our study, suggested the heterogeneity of this cell population. To our knowledge, this result has never been reported in the literature, but, to date, the origin and the function of this cell population remain controversial [31].

## 5. Conclusions

Altogether, these data point out the need for further longitudinal studies especially in goats. However, when working with outbred animal populations a high number of individual is required for sequential slaughtering due to interindividual variation, and thus only limited time points could be considered. Thus, experimental models should be developed to allow the monitoring of the histopathological changes and different immune mediators within the same animal in order to characterize finely the dynamic of the local and the peripheral mechanisms associated with the protective immune response. Moreover, the effect of the genetic status on globule leukocytes counts but not on the other inflammatory cells highlight the need for further studies on the functional activity of these cell populations.

## Figures and Tables

**Table 1:** Means of worm count in abomasal contents at 49 days postinfection for challenge 1 and 42 days postinfection for challenge 2.

	Challenge 1			Challenge 2			^5^ *P* value
	^1^R	^2^S	^3^ *P* value	^1^R	^2^S	^4^ *P* value	
^ 6^Male	234 ± 66	120 ± 147	0.49	203 ± 66	195 ± 65	0.93	0.81
^ 7^Female	241 ± 59	118 ± 72	0.19	234 ± 59	178 ± 59	0.51	0.45
^ 8^Immature male	5 ± 7	31 ± 8	0.65	15 ± 8	36 ± 9	0.21	0.02
^ 9^Immature female	19 ± 18	24 ± 22	0.56	46 ± 18	71 ± 18	0.53	0.05
^ 10^Female length	18.8 ± 0.5	21.0 ± 1.0	0.07	18.0 ± 5	18.2 ± 0.6	0.8	0.02

^1^R: resistant kids.

^2^S: susceptible kids.

^3^ *P* value: comparing means of worm counts in abomasal contents of resistant and susceptible kids after challenge 1.

^4^ *P* value: comparing means of worm counts in abomasal contents of resistant and susceptible kids after challenge 2.

^5^ *P* value: comparing means worm counts in abomasal contents after challenge 1 and challenge 2.

^6^Male: means of adults male worms in abomasal contents.

^7^Female: means of adults female worms in abomasal contents.

^8^Immature male: means of immature male worms in abomasal contents.

^9^Immature female: means of immature female worms in abomasal contents.

^10^Female length: means length (mm) of twenty percent of adult female worms in abomasal contents.

**Table 2:** Correlation coefficients between parasitological and physiological variables at slaughter.

	^ 1^FEC	^ 2^PCV	^ 3^Eosinophilia
^ 4^Male	0.295*	−0.31*	−0.57*
^ 5^Female	0.02	0.04	0.53*
^ 6^Immature male	−0.11*	−0.11*	0.18*
^ 7^Immature female	−0.33*	0.20*	−0.15*
^ 8^Female length	0.70*	−0.72*	−0.34*

*P < 0.0001.

^1^FEC: fecal egg counts (eggs per gram of feces).

^2^PCV: packed cell volume.

^3^Eosinophilia: blood eosinophils (number of cells per mL of blood).

^4^Male: means of adults male worms in abomasal contents.

^5^Female: means of adults female worms in abomasal contents.

^6^Immature male: means of immature male worms in abomasal contents.

^7^Immature female: means of immature female worms in abomasal contents.

^8^Female length: means length (mm) of twenty percent of adult female worms in abomasal contents.

**Table:** (a)

	Challenge 1		Challenge 2	
	^ 1^Eosi	^ 2^Mono	Eosi	Mono
^ 3^Resistant	2	3	2	2
^ 4^Susceptible	3	2	2	2
^ 5^Control	0	1	0	1

^1^Eosi: intensity of eosinophil infiltration in abomasal mucosa: (1) 1–8 cells/mm^2^; (2) 9–20 cells/mm^2^; (3) more than 20 cells/mm^2^.

^2^Mono: intensity of mononuclear cell infiltration in abomasal mucosa: (1) 1–8 cells/mm^2^; (2) 9–20 cells/mm^2^; (3) more than 20 cells/mm^2^.

^3^Resistant: resistant kids (*n* = 4 for challenge 1 and *n* = 6 for challenge 2) infected with 10,000 *H. contortus* infective larvae (L3) at day 0 postinfection.

^4^Susceptible: susceptible kids (*n* = 4 for challenge 1 and *n* = 6 for challenge 2) infected with 10,000 *H. contortus* infective larvae (L3) at day 0 postinfection.

^5^Control: noninfected kids (*n* = 2 for both challenges half resistant and half susceptible).

**Table:** (b)

	^ 1^Challenge 1	^ 1^Challenge 2
^ 2^Resistant	6.3 (1.6)	2.2 (0.2)
^ 3^Susceptible	2.7 (0.6)	0.9 (1.5)
^ 4^Control	0	0
*P* value	0.04	0.14

^1^Challenges 1 and 2: first and second challenges with 10,000 *H. contortus* L3.

^2^Resistant: resistant kids (*n* = 4 for challenge 1 and *n* = 6 for challenge 2) infected with 10,000 *H. contortus* infective larvae (L3) at day 0 postinfection.

^3^Susceptible: susceptible kids (*n* = 4 for challenge 1 and *n* = 6 for challenge 2) infected with 10,000 *H. contortus* infective larvae (L3) at day 0 postinfection.

^4^Control: noninfected kids (*n* = 2 for both challenges half resistant and half susceptible).
